# Sir Astley Cooper

**Published:** 1843-12

**Authors:** 


*Sir Astley Cooper.*—The *Medico-Chirurgical Review* thus terminates a review of Mr. Bransby B. Cooper's Memoir of this distinguished man:

"Having been acquainted with Sir A. Cooper for upwards of 35 years before his death—from having been one of his pupils—from having had frequent professional intercourse with him during the last twenty years of his life—from having read all his works, and heard a great deal of him from all quarters, professional and non-professional, we shall endeavour to draw up a succinct summary of the prominent features of his character.

RESUME.

"I. Sir A. was born and imbued with a remarkable vigour of body and activity of mind.

"II. AMBITION was his ruling passion.

"III. This ruling passion, conjoined with physical strength and mental energy, would have placed him at the head of any profession which chance or inclination assigned him. The combination which made him the leader in all juvenile sports, adventures, and dangers, would have made him a Napoleon or Wellington in war—a Pitt or a Peel in the Senate. It raised him to the summit of honors in Surgery.

"IV. Without being avaricious, much less sordid, he was fond of money—partly, perhaps, from the natural love of independence—partly as a means of gratifying his ambition, by the ability of procuring all things necessary for the attainment of his objects.

"V. His industry was never surpassed; and could only have been sustained by a Herculean frame like his, worked on by insatiable thirst of knowledge.

"VI. His temperance, in regard to wine and other stimulating drinks was, perhaps, unparalleled; but he fell into great errors, by the rapid manner in which he bolted his food, which was not half masticated.

"VII. For aught that appears to the contrary, the subject of our biography was as little open to the seductions of VENUS, as to the temptations of BACCHUS. Whether this was owing to constitutional temperament, moral discipline, or religious principles—or all three, we cannot now ascertain. The constant activity of mind and body in the pursuit of knowledge left him little time for sensual indulgences.

"VIII. In early life Mr. Cooper was a Radical—or something *more*—a Revolutionist. In mature age, Sir Astley was a Tory—or something *less*—a Conservative.

"IX. In religion, the association with Cline, Thelwall, and others of that school, in all probability led to FREE THINKING. In the intense application to medical studies, and during the turmoil of practice, we suspect that Sir Astley did not think much about the matter. His morals, however, were not merely unimpeachable, but of the highest order.

"X. In his intercourse with his professional brethren, he was always kind, considerate, and charitable towards their errors—adopting the Christian maxim

'To hide the faults we see.'

But in the bustle and hurry of miscellaneous prescription at his own house, Sir Astley was not always cautious enough to conceal or pass unnoticed, what he considered as MALA PRAXIS, or blundering treatment in others. In this respect, the late Dr. Baillie was a perfect model for imitation by the consulting physicians and surgeons of the metropolis and great provincial towns. Whatever he may have thought of the methodus medendi adopted by the practitioner who preceded him, he never let fall a syllable or the slightest hint or sign that he disapproved of the procedure previously pursued. Those patients, or rather IMPATIENTS, who appeal from their ordinary to the *consulting practitioner*, are very prone to misconstrue every word and even look of the latter into an indication that their case had been hitherto mistaken, and of course, mistreated. Every man of honor or honesty ought to be especially on his guard against fostering the prejudices of these malcontents, and thus injuring the character of the profession at large. We regret to say that we have known many of those who pride themselves on a high sense of moral rectitude, give way to this vile system of detraction, by which the noble science of medicine is wounded in its most vital parts, and degraded in its character. This detraction is even carried into the public prints, and too often becomes the subject of legal proceedings!

"XI. We need hardly observe that the subject of these memoirs was a prime favorite with the public, as well as with the faculty:—we may safely add, that he was not less so with himself. Few men had a better opinion of themselves, personal or professional, than Sir Astley Cooper. Durine a long acquaintance we never once saw him enter a drawing-room, and pass a mirror, without casting a furtive glance of inquiry, not unfrequently mixed with a smile of approbation at a fine figure that seemed to return the compliment *through* the pellucid glass. So in a professional point of point of view, Sir A. entertained so profound a respect for his own opinion, that he generally preferred it to the opinion of others, and therefore, in nine consultations out of ten, his word was law.

"XII. Truth, which is said to *lie* in a well, (though we could never see any lucid reason for the supposition), was held in such veneration by Sir Astley, that he never placed confidence in any man whom he found to deviate from that cardinal virtue. We have known him *embellish* an anecdote, a bon-mot, or a humorous incident; but we believe that he adhered to the rigid and naked truth on all other points.

"XIII. His devotion to science, and his indefatigable pursuit of knowledge, rendered it, perhaps, impossible that Sir Astley's mind could become susceptible of keen attachment or friendship to an individual. Intense exercise of the intellectual powers is little favorable to deep culture or centralization of the affections. Sir A. had numerous friends and still more numerous admirers, but it is doubtful whether he entertained any very ardent friendship himself for either one or the other.

"XIV. In respect to Love, his marriage in twelve months after the death of his wife, and when he had passed his "grand climacteric," offers a rather equivocal proof of his wisdom, whatever else it may indicate. True, he had precedents innumerable for this procedure; but precedents in love, law, and physic—especially in the *first* and *third*, are not always followed with perfect safety. It may be urged, on the other hand, that Sir Astley had a very *fair* excuse for matrimony, considering the personal attractions of Miss Jones—and that long courtships, at all times, but particularly after sixty years of age, are somewhat hazardous!

"XV. Sir Astley Cooper was a bold as well as dexterous operator. His ligatures on the carotids and aorta are sufficient illustrations. No man had a steadier hand, and a keener eye, or sharper knife—or made a cleaner cut than Sir A. He did not finesse, or fritter away his time in dividing fibre after fibre, even in somewhat ticklish dissections. He generally "went the whole hog"—to the root of the evil, with a few strokes of the scalpel, while his cheerful and animated countenance supported the confidence of the bleeding victim!*

"XVI. Although more than half of Sir Astley's morning practice at home, was what is called purely *medical*, yet he did not keep pace on all points with the modern improvements in pathology, semeiology, or therapeutics. Auscultation was introduced into this country in the year 1818, when Sir Astley was about the age of 50, and, considering his zeal and energy, it is wonderful that he did not cultivate this, the most scientific branch of our art. The stethoscope apart, however, his diagnosis of organic diseases of internal as well as external parts, was remarkably correct, and did great credit to his acumen and judgment.

"XVII. Taking him all in all, it is very unlikely that the professional world will ever see among them such a master mind and master hand again, as Sir Astley Cooper. Not that his genius and talents will not find equals in every successive generation, but that the general elevation of medical education will prevent any individual in future from soaring high over the heads of his cotemporaries. Among a race of *pigmies*, there may be occasionally a full grown HOMO; but among men how rarely do we see a giant?

"XVIII. It is an ungrateful task to find fault with the last act of a great man. But we are constrained to do so in the present case. We acknowledge, with a noble Lord, that 'every man has a *right*, (a legal one), to do as he likes with his own.' But LAW is not always EQUITY; and the rich man

---

* "We were under the hands of Sir A. Cooper and Mr. Guthrie, in some trying operations. It is not for us to say which we would prefer, *now* that the hand of one is cold and powerless. But we may state that these eminent masters of their art *were*

——————'Arcades ambo
Et *secare* pares, et *re*-secare parati.'

"Apropos, as to the sensations experienced during a cutting operation. Not one in 500 surgeons know what they are—luckily for themselves! We had formed a very erroneous opinion of them before we became acquainted with the scalpels of the above-mentioned surgeons. They are not pain, but a *burning* sensation, as though a fine stream of boiling lead were jetting on the parts."—*Rev.*

who bequeaths his wealth more in accordance with the *former* than the LATTER, is not entitled to post-obit praise on this point. The last and testamentary act of Sir Astley Cooper proves the truth of our assertion at the beginning:—that *ambition* was his *ruling passion!* He has left the hard-earned masses of forty years' toil and privation to support a title and incorporate his name with the aristocracy, which, in early life, he despised, while he has left a paltry thousand to his eleve—his nephew—and the most honorable, amiable, and talented of all his relations!!

CONCLUSION.—"We cannot close this article without saying a few words as to the biography and the biographer. We think Mr. B. Cooper has been rather severely handled by some of his critics. They aver that the biography is needleesly minute, and badly arranged. The work is certainly not conspicuous for the LUCIDUS ORDO; nor is it remarkable for its concentrativeness. But it is the very nature of a biographer to be *minute.* We hunt after the little sayings and doings of a great man, with much more curiosity than after the main and more public acts of his life.

'Nihil est aliud magnum quam multa minuta.'

"Nothing was ever more minute, and gossipy than Boswell's life of Johnson, and yet to that very quality was half its popularity owing. Some critics say that the work is badly written. We do not think so;—and we have read every page of it. It is better written than some of the critiques on it. We do not deny that it is diffuse, and not well arranged; but we are of opinion that the heterogeneous materials from which the narrative of the biography was constructed, would have puzzled the ablest and the most practised writer.

"The introduction of the RESURRECTION-SCENES is another charge brought against the biographer by the squeamish sentimentalists of the day. But what was of vast importance to Sir Astley, and indeed to the whole of the profession at that time, should not be passed unnoticed now that such scenes are swept away by legal enactments.

"We are of opinion, however, that in a future edition (and many will be called for), Mr. Cooper may usefully compress the matter, making up for this compression, by an extension of those excellent moral and professional remarks and counsels which are scattered through the volumes as they stand."

